# Discovery of key biomarkers in tourette syndrome by network pharmacology

**DOI:** 10.3389/fphar.2024.1397203

**Published:** 2024-09-10

**Authors:** Jiali Zhao, Xiaohong Bai

**Affiliations:** ^1^ Liaoning University of Traditional Chinese Medicine, Shenyang, China; ^2^ Harbin Hospital of Traditional Chinese Medicine, Harbin, Heilongjiang, China; ^3^ Affiliated Hospital of Liaoning University of Traditional Chinese Medicine, Shenyang, China

**Keywords:** tourette syndrome, Yangxue Xifeng decoction, network pharmacology, immune infiltration, LASSO regression and SVM algorithm

## Abstract

**Background:**

Yangxue Xifeng Decoction (YXD) has been utilized in clinical settings for the treatment of Tourette Syndrome (TS). However, the action mechanism of YXD needs further research.

**Methods:**

The ingredients and targets of YXD were identified via database searches and then constructed an active ingredient-target network using Cytoscape. Pathway enrichment analysis was performed via Gene Ontology (GO) and Kyoto Encyclopedia of Genes and Genomes (KEGG). The core genes were determined by LASSO regression and SVM algorithm. Additionally, we analyzed the immune infiltration. The signaling pathways associated with core genes were investigated through KEGG and GO. We predicted the transcription factors using “RcisTarge”.

**Results:**

127 active ingredients of YXD and 255 targets were obtained. TNF and the IL-17 signaling pathway were the main pathways. *OPRM1* and *VIM* were screened out as core genes, which were associated with the immune infiltration. The signaling pathways involved in *OPRM1* and *VIM* were enriched. Furthermore, remarkable correlation was found between *OPRM1* and *VIM* levels and other TS-related genes such as *MAPT* and *MAPT*.

**Conclusion:**

*OPRM1* and *MAPT,* and the signaling pathways are associated with TS. YXD exerts its therapeutic TS through multi-component and multi-targets including immune infiltration.

## 1 Introduction

Tourette syndrome (TS), also known as tourette disorder, is a neurodevelopmental condition characterized by persistent vocal and motor tics that cause distress and impair daily functioning. (First et al., 2012). Estimates suggest that TS affects between 0.1% and 1% of children. (Nilles et al., 2023; Efron and Dale, 2018). The presence of psychiatric comorbidities, such as attention deficit-hyperactivity disorder (ADHD) and obsessive-compulsive disorder (OCD), can have a more significant impact than the tics themselves. (Eapen et al., 2016a; Ogundele and Ayyash, 2018; Cavanna et al., 2020). Research indicates that a large proportion, up to 85%, of children with TS also experience other neurodevelopmental or mental health conditions. (Kattner, 2022). Additionally, individuals with TS commonly exhibit symptoms of anxiety, behavior problems, learning difficulties, sleep disturbances, impaired social interaction, and sensory processing challenges. (Nilles et al., 2023; Lin et al., 2022; Set and Warner, 2021). Numerous studies consistently show that both children and adults with TS have a reduced quality of life. (Eapen et al., 2016b; Gilbert et al., 2024).

**TABLE 1:** Genes information.

Full official gene name	Description	Gene ID number	MIM (Mendelian inheritance in Man) number	Chromosome location
OPRM1	opioid receptor mu 1 [*Homo sapiens* (human)]	4,988	600018	Chromosome 6, NC_000006.12 (154010496.154246867)
VIM	vimentin [*Homo sapiens* (human)]	7,431	193060	Chromosome 10, NC_000010.11 (17228241.17237593)
MAPT	crotubule associated protein tau [*Homo sapiens* (human)]	4,137	157140	Chromosome 17, NC_000017.11 (45894554.46028334)
MAPK1	mitogen-activated protein kinase 1 [*Homo sapiens* (human)]	5,594	176948	Chromosome 22, NC_000022.11 (21759657.21867680, complement)
CIC	capicua transcriptional repressor [*Homo sapiens* (human)]	23152	612082	Chromosome 19, NC_000019.10 (42268530.42295796)
DLGAP3	DLG associated protein 3 [*Homo sapiens* (human)]	8,512	611413	Chromosome 1, NC_000001.11 (34865436.34929650, complement)
KIR2DL3	killer cell immunoglobulin like receptor, two Ig domains and long cytoplasmic tail 3 [*Homo sapiens* (human)]	3,804	604938	Chromosome 19, NC_000019.10 (54738513.54753052)
KIR2DL1	killer cell immunoglobulin like receptor, two Ig domains and long cytoplasmic tail 1 [*Homo sapiens* (human)]	3,802	604936	Chromosome 19, NC_000019.10 (54769793.54784322)
TNFRSF13B	TNF receptor superfamily member 13B [*Homo sapiens* (human)]	23495	604907	Chromosome 17, NC_000017.11 (16939081.16972118, complement)
TNFSF9	TNF superfamily member 9 [*Homo sapiens* (human)]	8,744	606182	Chromosome 19, NC_000019.10 (6531026.6535924)
CD276	CD276 molecule [*Homo sapiens* (human)]	80381	605715	Chromosome 15, NC_000015.10 (73683944.73714514)
HLA-DPA1	major histocompatibility complex, class II, DP alpha 1 [*Homo sapiens* (human)]	3,113	142880	Chromosome 6, NC_000006.12 (33064569.33080748, complement)
CXCR4	C-X-C motif chemokine receptor 4 [*Homo sapiens* (human)]	7,852	162643	Chromosome 2, NC_000002.12 (136114349.136118149, complement)
CXCR3	C-X-C motif chemokine receptor 3 [*Homo sapiens* (human)]	2,833	300574	Chromosome X, NC_000023.11 (71615919.71618511, complement)
NAALAD2	N-acetylated alpha-linked acidic dipeptidase 2 [*Homo sapiens* (human)]	10003	611636	Chromosome 11, NC_000011.10 (90131699.90192894)
FLT3	fms related receptor tyrosine kinase 3 [*Homo sapiens* (human)]	2,322	136351	Chromosome 13, NC_000013.11 (28003274.28100576, complement)

The pathophysiology is of TS remains unclear, including various defects in neurobiology caused by inherited genetics, surrounding environment, infections, and psychosocial elements. (Kattner, 2022; Lin et al., 2022; Johnson et al., 2023; Billnitzer and Jankovic, 2020). Abnormalities in the pathways that connect the cerebral cortex and basal ganglia have been identified as causing neuronal disinhibition in the motor and limbic systems. (Cavanna et al., 2020; Ramkiran et al., 2019). Evidence also suggests that the sensory limbic and executive corticostriatal loops play a role in this process. (Felling and Singer, 2011). In terms of biochemistry, multiple neurochemical pathways are implicated, involving imbalances in amine neurotransmitters and other signaling molecules such as endogenous opioid peptides. (Szejko et al., 2022). Besides, TS appears to have a strong genetic foundation, with an inheritance pattern most likely controlled by multiple genes. (Lin et al., 2022; Domènech et al., 2021). The diagnosis and clinical management of the disease are significantly influenced by the immune microenvironment which consists of immune cells, inflammatory and growth factors, extracellular matrix and etc. (Tsetsos et al., 2024) In some cases, abnormalities in immune activation can lead to inflammation, which appears to play a pathogenic role. (Lin et al., 2022; Martino et al., 2020).

The treatment approach for Tourette Syndrome (TS) varies depending on the type and severity of the injury sustained by the patient. (Hsu et al., 2021a; Leckman et al., 2005). Acceptance, understanding, education and are typically enough for most patients. However, psychological or pharmacological treatments might become crucial in more serious situations. (Pringsheim et al., 2019a; Pringsheim et al., 2019b). It is important to note that medication is still the main treatment of TS. (Seideman Mf Fau - Seideman and Seideman, 2020). However, current drugs for TS are primarily psychiatric medications, which often have significant side effects. (Chen et al., 2020). The US Food and Drug Administration (FDA) has approved some medications to manage tics in TS, all of which are antipsychotics that act on the D2 receptor. Despite their effectiveness, both first- and second-generation D2 receptor antagonists are associated with side effects such as somnolence, dysphoria, and the risk of drug-induced movement disorders like dystonia, akathisia, withdrawal dyskinesias, and rarely, tardive dyskinesia. (Pringsheim et al., 2019b; Buse et al., 2013). Moreover, the use of D2 receptor antagonists in TS is limited due to the risk of increased weight gain, elevated prolactin, dyslipidemia, and hyperglycemia, including diabetes. However, one of the main challenges in developing drugs for TS is the lack of clear therapeutic targets for its pathogenesis.

Traditional Chinese Medicine (TCM) offers potential alternative treatments for TS. Yangxue Xifeng Decoction (YXD), a herbal formula composed of 10 herbs, has been shown to significantly improve clinical symptoms in TS patients in China. Nevertheless, the therapeutic targets and mechanisms of Chinese medicines (CM), including YXD, are not yet clear due to the complexity of their components. Network pharmacology presents a novel method for examining the correlation between medications and illnesses. By combining multidirectional pharmaceutical biology, systems biology, computer science, and bioinformatics, network pharmacology allows for the systematic investigation of molecular regulatory mechanisms. (Zhou et al., 2020). The multi-target and multi-drug model enables the construction of more efficient networks, which helps in understanding the complex composition and multiple therapeutic targets of CM formulas. (Luo et al., 2020; Shang et al., 2023). Network pharmacology offers an evidence-based approach for researches of clinical efficacy and quality control on CM formulas. (Luo et al., 2020; Zhou et al., 2020; Niu et al., 2022).

In present study, the material basis and action mechanism of YXD for TS, and the potential biomarkers for TS were investigated. Various network pharmacology methods were performed to predict the Targets and Pathways of YXD for TS. SVM algorithm analyses and LASSO regression were performed for identification of TS core genes. Additionally, the relationship between immune cells and central genes, signaling pathways, key genes transcriptional regulation, and genes that synergize with key genes were analyzed.

## 2 Materials and methods

### 2.1 Predicting the targets and pathways of YXD for TS

The medicinal ingredients of all the herbs involved in TXD were obtained based on TCMSP database. The YXD ingredients were screened out based on pharmacokinetic information including drug-likeness (DL) and oral bioavailability (OB). DL was evaluated using Lipinski’s Rule of Five, with compounds meeting at least 4 criteria considered to have good drug-likeness, and OB was assessed using the threshold of ≥30%. These parameters were chosen as they are widely accepted indicators of a compound’s potential as an orally active drug in humans. (Liu et al., 2021). The TS-related targets were looked up based on OMIM, Genecard, and GEO databases. The pathological targets of TS, and the possible drug targets of YXD ingredients were integrated by Venn diagrams, and the intersecting proteins were used as the potential target proteins of YXD for the treatment of TS. Herb-compound-target network diagrams were visualized by using Cytoscape.

### 2.2 Data acquisition from GEO database

The data of gene expression from the TS dataset GSE30470 was downloaded from the Gene Expression Omnibus (GEO) database with the annotation platform GPL570, containing control (n = 13) and disease (n = 8) groups, from which 255 TS-related target proteins were screened as biomarkers.

### 2.3 LASSO and SVM-RFE model

TS diagnostic markers were categorized using SVM algorithms and LASSO logistic regression. We performed LASSO analysis using the “glmnet” software package. In addition, the selected biomarkers were analyzed for TS diagnosis by using the “e1071” software package to build a SVM model.

### 2.4 Analysis of immune infiltration

In this study, we utilized the ssGSEA algorithm to assess the impact of gene expression on immune cell infiltration levels within each sample. Furthermore, the corrplot package was employed for examining the relationships between different immune cell types and conducting a comprehensive analysis of immune cell interactions. Additionally, Pearson correlation analysis was carried out to investigate the association between gene expression levels and immune cell content.

### 2.5 Gene set enrichment analysis

To conduct a comprehensive plagiarism check, we utilize the Gene Set Enrichment Analysis (GSEA) technique. Our analysis entails the utilization of a predefined gene set, in which genes are organized according to their degree of differential expression across two distinct group. The criteria for selecting these sets is FDR <0.05 and containing at least 10 genes from our input list. The primary objective is to determine if the predefined set of genes exhibits enrichment either at the upper or lower strata of this ranked list. This investigation delves into the molecular mechanisms underlying the core genes within the two patient groups. This study explores the molecular mechanisms that underlie the core genes in the two sets of patients. GSEA enables us to gain important insights by examining the differences in signaling pathways between the groups with high and low expression.

### 2.6 Transcriptional regulation analysis of key genes

The predictions regarding transcription factors were made using the R package “RcisTarget.” Within this package, algorithms are utilized that depend on motifs for the predictive analysis. Assessment of the motifs was conducted using the normalized enrichment score, which is contingent upon the total number of motifs in the database. Additionally, inference of additional annotation files was made based on motif similarity and gene sequences. To evaluate the overexpression of each motif on a gene set, the initial step involved computing the area under the curve (AUC) for every pair of motifs. This calculation was performed by organizing the gene set in the order of its motifs to generate the recovery curve. By considering the AUC distribution of all motifs within the gene set, the normalized enrichment score (NES) for each motif was determined.

### 2.7 miRNA analysis

In order to conduct a thorough investigation into whether specific miRNAs in crucial genes control the transcribing or breaking down of those genes, we accessed miRNAs associated with key genes from the mircode database. Subsequently, we used cytoscape software to create visual representations of the miRNA networks within these genes.

### 2.8 Statistical analysis

The R software was used to conduct all statistical analyses. The difference was considered significant when *p* < 0.05.

## 3 Results

### 3.1 Predicting the Targets and Pathways of YXD for TS

**FIGURE 1:**
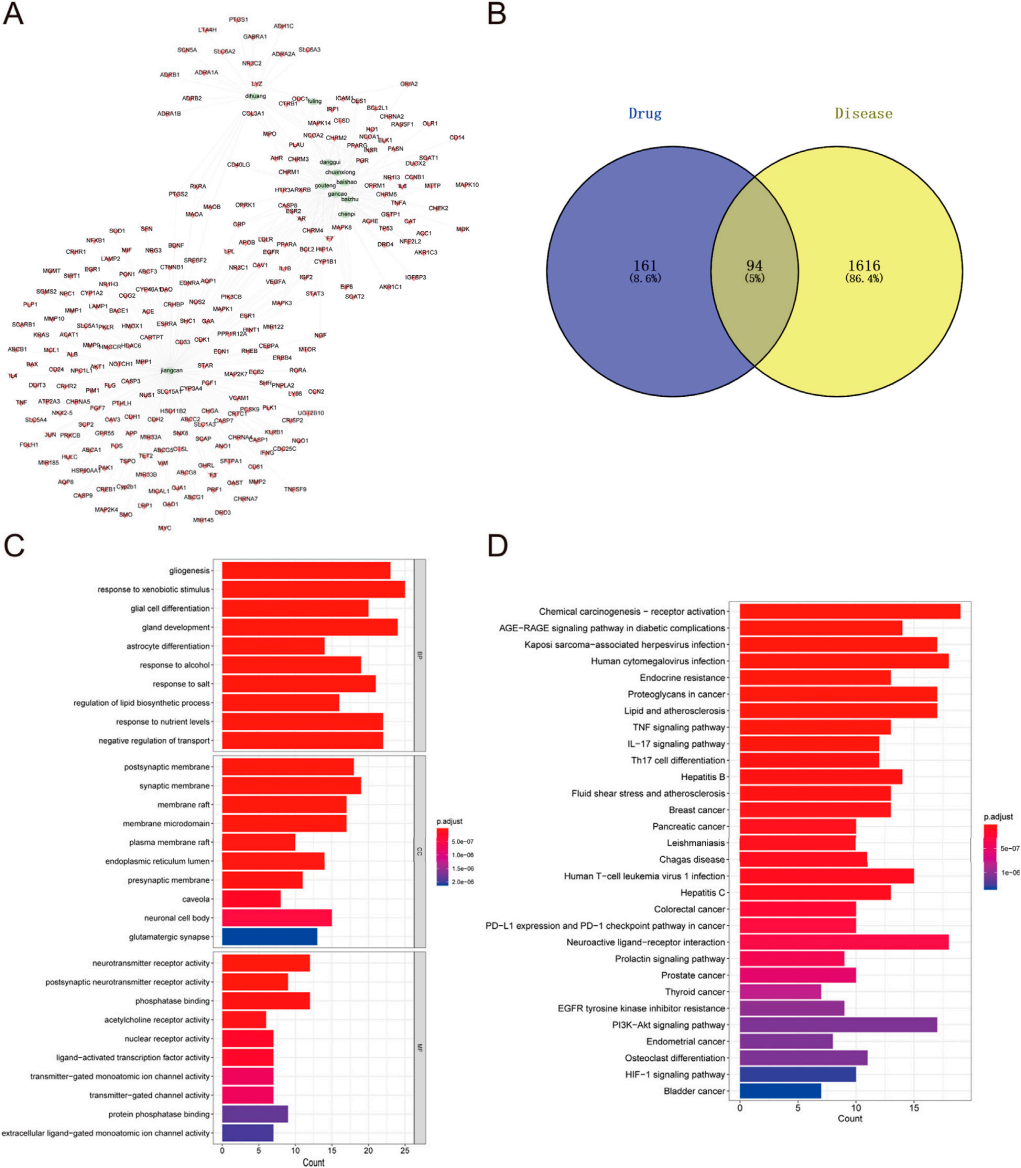
**(A)** Herb-compound-target network of Yangxue Xifeng Decoration. Green elliptic represens 10 herbs in the formula. The red quads stand for the active ingredients of all of 10 herbs. **(B)** Venn diagram of comen targets of active ingredients in modified Yangxue Xifeng Decoration and Tourette symdrome. **(C)** GO analysis bubble diagram for the intersecting target genes. **(D)** Bubble diagram of KEGG analysis for the intersecting genes.

**FIGURE 2:**
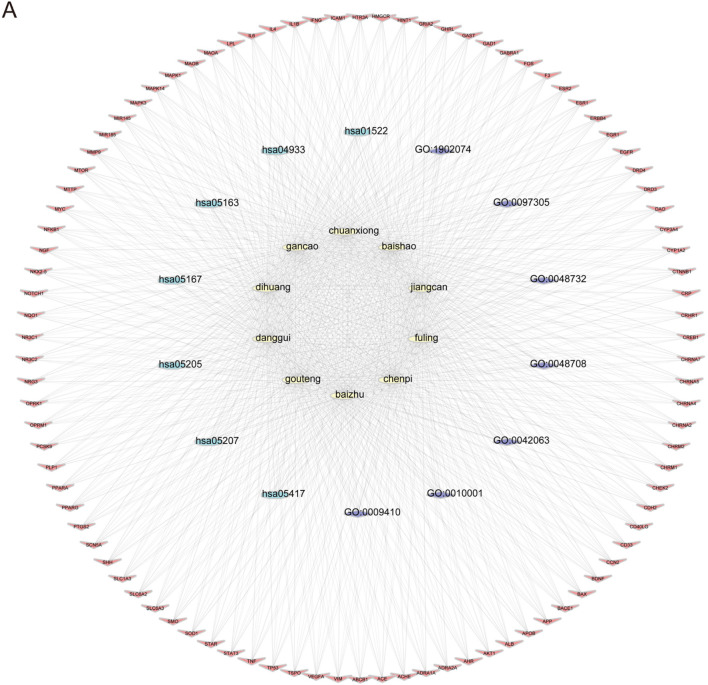
**(A)** Herb-pathway-target network of Yangxue Xifeng Decoration. Yellow elliptic represent 10 herbs, blue and purple circles represent pathways, and red quads represent target protein.

To explore the potential targets and action mechanisms of YXD in treating TS, we applied the network pharmacology method to screen the targets of YXD and predict the signaling pathways involved in its therapeutic effects. Firstly, we searched the TCMSP for identification of all ingredients and related targets of the 10 herbs included in YXD. Then, we screened the active ingredients of YXD complex based on OB and DL parameters. As a result, we obtained a total of 127 active ingredients and 255 drug targets (8.6%) (Figure 1B), with gene names added for all targets. Subsequently, we used the Gene Cards database to extract genes with a Relevance score greater than 5, and the OMIM database to obtain a total of 1,710 relevant targets (86.4%) for TS after removing duplicates (Figure 1B). Through taking the intersection of these disease targets and 255 drug targets, we obtained 94 intersecting targets (5%) (Figure 1B). The action relationships of the 10 TCMs with their action targets were illustrated using network diagrams in Cytoscape (Figure 1A). Using the R package “clusterprofiler,” we performed GO enrichment and KEGG pathway analysis of 94 intersecting targets. The GO enrichment results revealed that the main pathways involved were gligogenesis, response to xenobiotic stimulus, glial cell differentiation, gland development, astrocyte differentiation, response to nutrient levels, response to alcohol, membrane raft, synaptic membrane, postsynaptic membrane, membrane microdomain, and etc. (Figure 1C). The KEGG results showed some significant pathways involved in the genes were chemical carcinogenesis-receptor activation, IL-17 signaling pathway, TNF signaling pathway, human cytomegalovirus infection, Kaposi sarcoma-associated herpesvirus infection, AGE-RAGE signaling pathway, and etc. (Figure 1D). Finally, we visualized the role of herbal components in relation to the targets and the regulatory networks of the pathways though depicting Herb-pathway-target network of YYXF decoction by Cytoscape software. (Figure 2).

### 3.2 Identification of core genes related to TS

**FIGURE 3:**
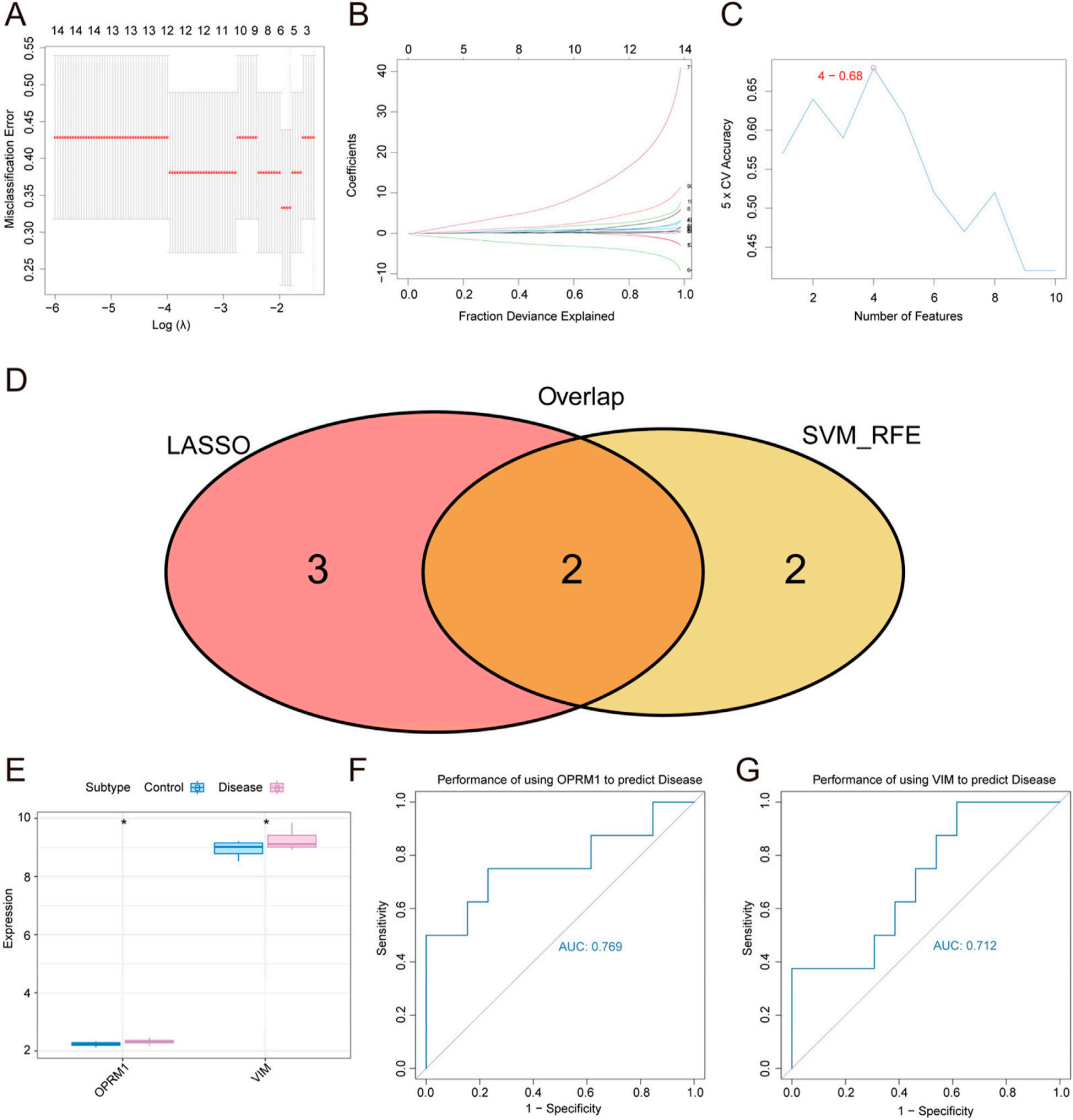
Screening of diagnostic markers for Tourette Syndrome by LASSO regression and SVM algorithms. **(A)** The misclassification error of the analysis of jackknife rates. **(B)** LASSO coefficient profiles of the genes meeting the prognostic criteria initially. **(C)** Number of features versus 5 × CV accuracy plot. **(D)** Genes venn diagram from LASSO and SVM-RFE methods. Two conmen hub genes screened out were *OPRM1* and *VIM*. **(E)** The expression difference of *OPRM1* and *VIM* in disease marked in red and control groups marked in blue. **(F)** ROC curves for evaluating the accuracy of performance of using the *OPRM1* gene to predict disease. **(G)** Using ROC curves to evaluate performance accuracy of disease prediction by *VIM* gene. *p* < 0.05 denoted statistical significance.

In order to screening out diagnostic markers for TS, we conducted a feature screening process to identify core genes related to TS as biomarkers, using LASSO regression and SVM algorithm. In Figure 3A, the analysis of misclassification errors in jackknife rates was demonstrated. Figure 3B displayed the profiles of LASSO coefficients for genes that initially fulfilled the prognostic criteria. Number of features versus 5 × CV accuracy plot was shown in Figure 3C. As a result, from the all of 96 intersecting genes obtained in the previous step, the LASSO regression screened out 5 characteristic genes in TS. Besides, the results of SVM algorithm help identified 4 characteristic genes in TS. The intersection of the two parts of genes from two methods resulted in 2 core genes, namely, *OPRM1* and *VIM*, which were further study in our subsequent study (Figure 3).

To validate the correlation between two core gens of *OPRM1* and *VIM* and TS, the expression difference of *OPRM1* and *VIM* between the normal control and TS disease groups were analyzed. The results revealed a marked variation in expression between the two groups. The levels of *OPRM1* and *VIM* expression were notably elevated in the TS disease groups compared to the normal control group. (Figure 3E). These results implied that there was a correlation between the two core genes and TS disease. To further explore the function of *OPRM1* and *VIM*, we evaluated the predictive efficacy of the core genes using ROC curves. These findings showed that the AUC values for *OPRM1* and *VIM* were 0.769 (*OPRM1*-AUC) and 0.712 (*VIM*-AUC), respectively, indicating their potential of *OPRM1* and *VIM* for predicting the development of TS (Figures 3F,G).

### 3.3 Immune infiltration analysis

**FIGURE 4:**
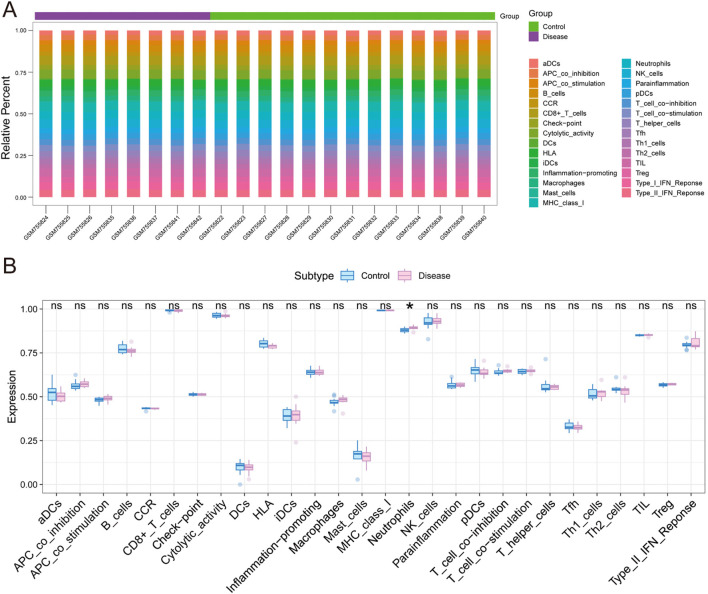
The analysis of immune infiltration in Tourette syndrome and control groups. **(A)** The box-plot indicates the varying proportions of distinct immune cells. **(B)** The heat map summarizies the immune infiltration score in Tourette syndrome and control groups. The immune infiltration difference in Tourette syndrome (red) and control groups (blue). (statistical significance indicated by *p*-values <0.05).

In order to further investigate the potential mechanisms of the core genes *OPRM1* and *VIM* in influencing TS progression, we conducted an analysis of immune infiltration in a TS dataset, comparing TS patients to normal controls. The box-plot diagram depicts the varied percentage of diverse immune cells, while the heat map encapsulates the immune infiltration score comparison among TS and normal group (Figures 4A,B). These findings suggest a noteworthy increasing in Neutrophil levels within the TS disease cohort in contrast with the normal group. There other cell types including NK-cells and Th1/2 cells, etc., showed no significant difference.

**FIGURE 5:**
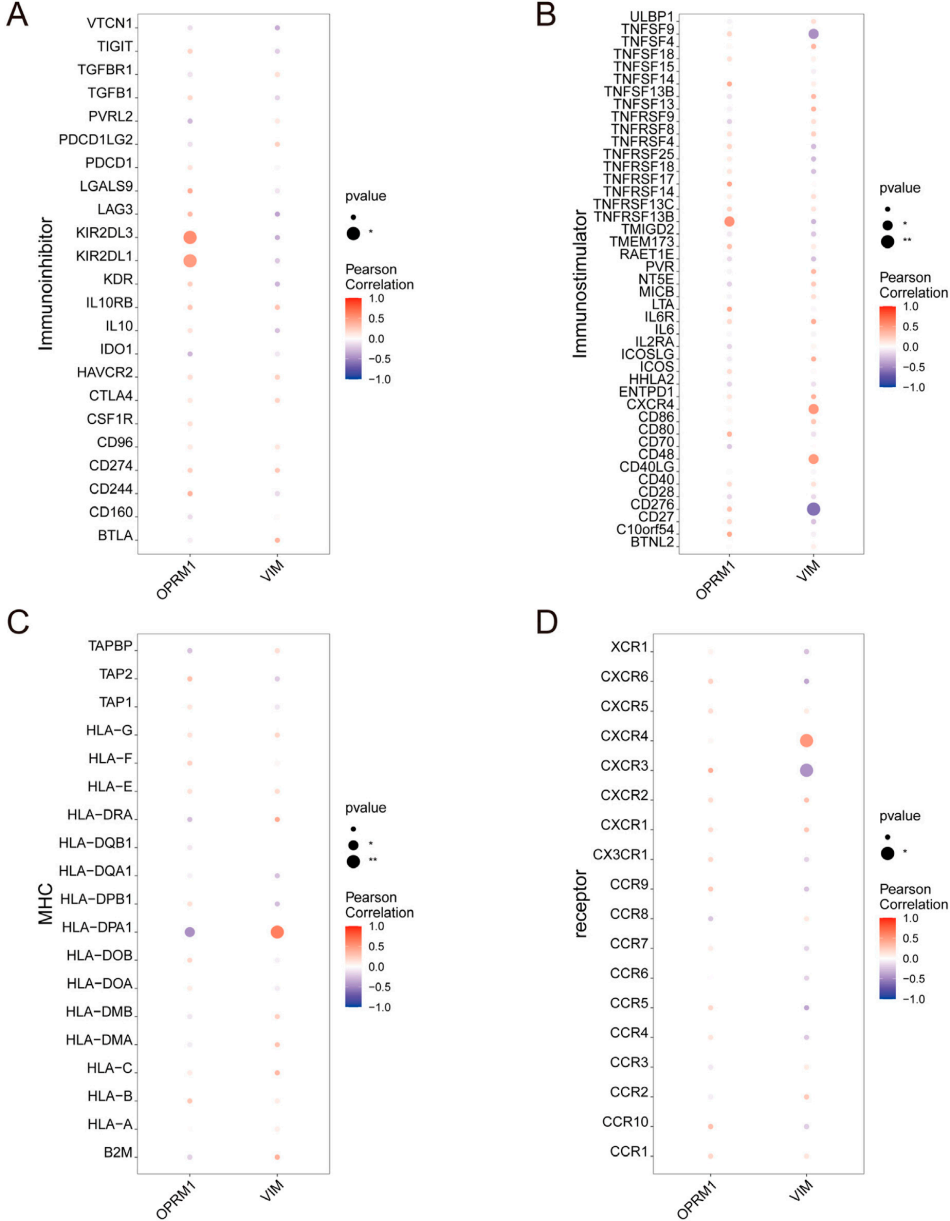
The relationship between core genes and immune cell permeation. Connections between characteristic genes and the level of infiltration. l including immune inhibitor **(A)**, immune stimulator **(B)**, MHC **(C)**, and receptor **(D)**.

**FIGURE 6:**
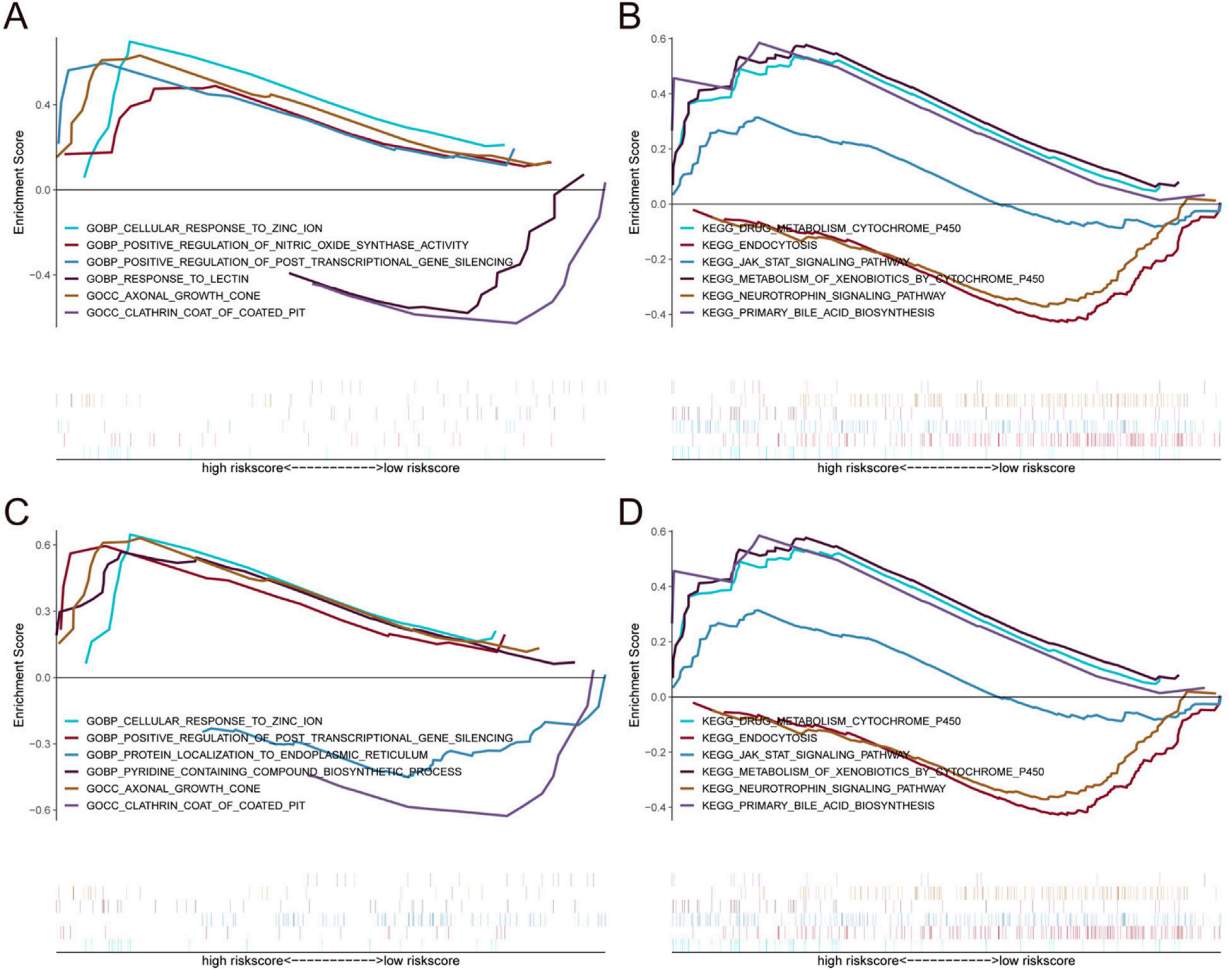
Enrichment analysis of pathway and gene ontology (GO) associated with core genes. **(A, B**) Gene Set enrichment analysis of *OPRM1*. **(C, D)** Gene Set Enrichment Analysis of *VIM*.

To further investigate the correlation between *OPRM1*, *VIM* and immune cells, based on the TISIDB database, we analyze the correlation between *OPRM1*, *VIM* and various immune factors. The results indicated that the immune inhibitors *KIR2DL3* and *KIR2DL1* were positively correlated with *OPRM1* (Figure 5A). *TNFRSF13B*, an immune stimulator, showed the highest positive correlation with *OPRM1* (Figure 5B). Additionally, Moreover, there was a positive correlation between *VIM* and immune stimulators CXCR4 and CD48. Conversely, *VIM* was negatively correlated with *TNFSF9* and *CD276* (Figure 5B). In terms of MHC, *OPRM1* was negatively correlated with *HLA-DPA1* but a positively correlated with *VIM* (Figure 5C). Furthermore, the *CXCR4* receptor exhibited mostly positive correlations with *VIM*, while *CXCR3* displayed predominantly negative associations with *VIM* (Figure 5D). These results indicated that *OPRM1* and *VIM* were intricately associated with infiltration of immune cells and may functioned to regulate immune microenvironment. Consequently, we propose that *VIM* and *OPRM1* are linked to the extent of immune infiltration and could potentially serve as crucial regulators of the immune microenvironment. To further explore the signal pathways related with *OPRM1* and *VIM*, we performed the pathway enrichment analysis and gene ontology (GO) associated with core genes (Figure 6). The results suggested *OPRM1* and *VIM* could function to regulate immune infiltration via multiple signal pathways.

### 3.4 Mechanisms of transcriptional regulation of key genes

**FIGURE 7:**
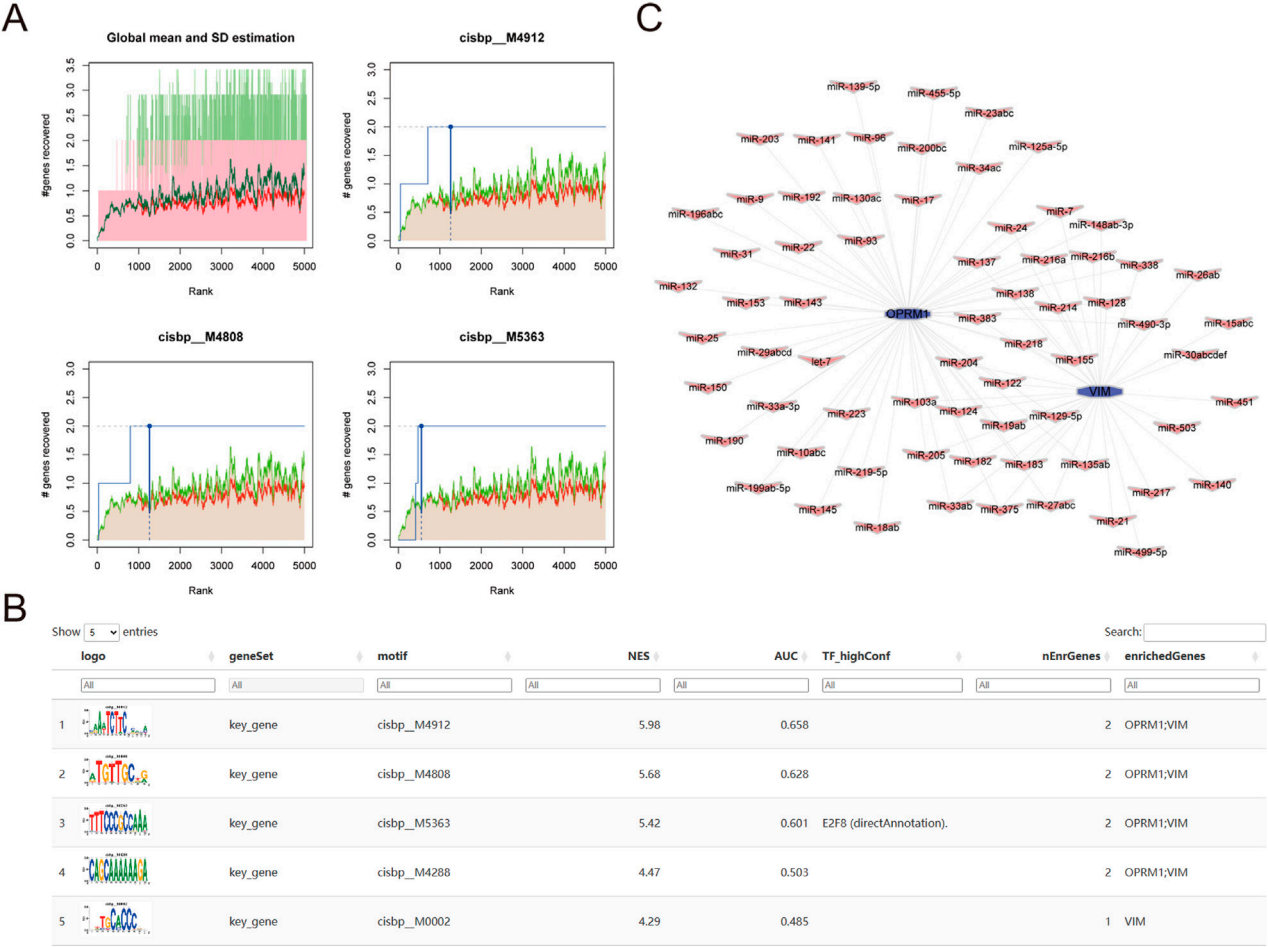
Analysis of core gene transcriptional regulation. **(A)** Three motifs with the highest AUC values. The average of the recovery curve was marked in red, mean + Std was marked in green, the recovery curve of the current motif was marked in blue. **(B)** Illustration of the motif with the highest AUC values, including TF_highConf (transcription factors), AUC (area under the curve), and NES (standardized enrichment score). **(C)** core genes miRNA networks, purple for the core genes of *OPRM1* and purple for miRNA.

To identify possible transcriptional regulatory mechanisms for the two core genes, R package “RcisTarget” was utilized to predict the transcription factors. A motif transcriptional regulation analysis of *OPRM1* and *VIM* was performed. Our findings revealed that both hub genes were controlled by the same transcription factors and other shared regulatory processes. Our findings revealed that both hub genes could be regulated by identical transcription factors and other common regulatory mechanisms. Utilizing the cumulative recovery curve (Figure 7A), motif-TF annotation, and selection analysis results of noteworthy genes, the enrichment analysis for transcription factors unveiled that the motif cisbp__M4912 had the top normalized enrichment score (NES: 6.92). This motif was enriched in the 2 core genes with a normalized enrichment score (NES) of 5.98. The motifs enriched in the 2 core genes and the associated transcription factors were presented in Figure 7B, which demonstrated the highest motif enrichment of AUC. Furthermore, we utilized the mircode database to search for non-coding RNA networks related to the two key genes. Our analysis predicted 59 mRNA-miRNA pairs related to *OPRM1* and 34 mRNA-miRNA pairs related to *VIM*. The miRNA networks of the core genes were visualized using Cytoscape (Figure 7C).

### 3.5 Predicting genes synergizing with key genes

**FIGURE 8:**
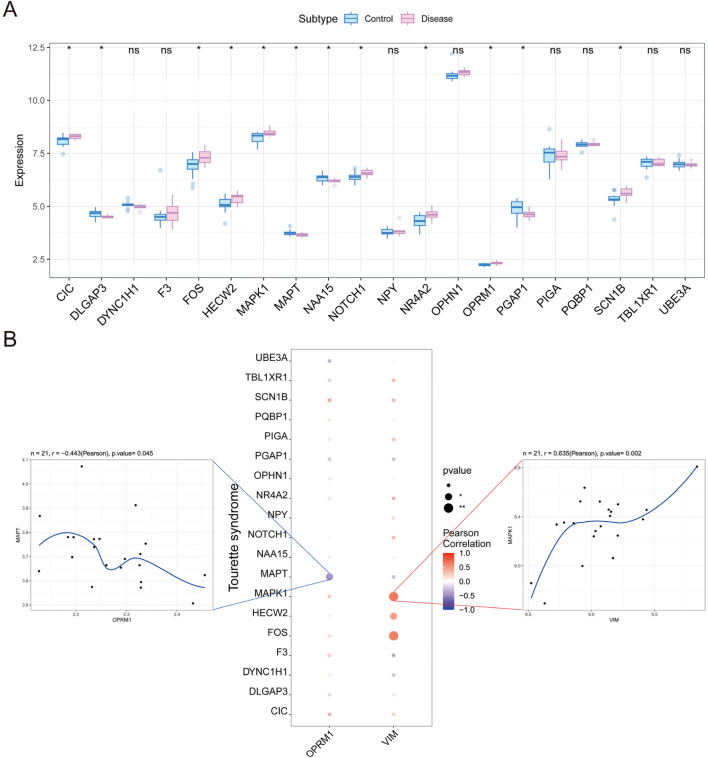
Analysis of correlation between the expression of core genes and other genes in Tourette syndrome. **(A)** The difference in other gene expression between tourette syndrome marked in red and controls marker in blue. **(B)** Relationships between other genes and the level of infiltration including immune inhibitor and immune simulator. Correlations between other genes and the hub gene including *OPRM1* (Left) and *VIM* (Right). *OPRM1* showed a significant negative correlation with *MAPT* (r = −0.443), whereas *VIM* demonstrated a significant positive correlation with *MAPT* (r = 0.63). *p* < 0.05 denoted statistical significance.

To identify genes that interact with core genes in influencing the progression of TS disease, we conducted a correlation analysis among the expressions of *OPRM1*, *VIM* and other genes related with TS. Initially, we obtained TS-related disease genes from Gene Cards data to compile a list of TS-related genes. Then, the expression differences of these genes between TS and normal controls were analyzed. The results indicated notable variation in the expression of *CIC*, *DLGAP3*, *MAPT*, and *MAPT* between the two groups (Figure 8A). Furthermore, we observed strong associations between the expression levels of *OPRM1*, *VIM* and other numerous TS-related genes (Figure 8B). Notably, *OPRM1* negatively correlated with *MAPT* (r = −0.443). Comparatively, *VIM* showed a positive correlation with *MAPT* (r = 0.63) (Figure 8B).

## 4 Discussion

Although TS is usually considered non-life threatening, it can significantly impact their social interactions and physical and mental health, due to the presence of tics and associated comorbidities. (Eapen et al., 2016a). However, the insufficient comprehension of the pathological mechanisms of TS led to less than ideal therapy outcomes. (Hartmann and Worbe, 2018). The management strategy entails addressing the symptoms that result in impairment, which may necessitate psychological and/or pharmacological intervention. (Billnitzer and Jankovic, 2020; Seideman Mf Fau - Seideman and Seideman, 2020). Antipsychotics, the solitary approved category of pharmacotherapy for tic suppression, possess an undesirable risk profile associated with weight gain, metabolic irregularities, electrocardiogram (ECG) modifications, and movement disorders. (Kim et al., 2018). Developing knowledge of the biological basis of TS will promote TS treatments instead of just symptom management. The precise pathophysiology of TS still remains elusive. Thus, treatment approaches often rely on empirical methods. (Chou et al., 2023).

There are various strategies in Traditional Chinese medicine (TCM) about tics treatment, but almost all the traditional Chinese strategies agree that the root cause lies in liver wind. YXD have specific effects in TCM theory, effectively alleviating symptoms such as muscle twitching, vocal tics, and obscene speech throughout the body. Besides definite efficacy, TCM has few side effects and drug dependence. (Xiao and Tao, 2017). However, lack of a clear material basis and action mechanism for YXD hinders the wider clinical application. In this study, we utilized drug target and disease target databases to identify that all active ingredients of YXD have 96 targets related to TS, confirming its multi-component and multi-target action. Searching for key genes associated with TS aims to identify biomarkers for accurate diagnosis. Currently, diagnosing the tic disorder relies on clinical history and observation, as there are no definitive laboratory tests or medical markers available. (Liu et al., 2020). This poses a challenge for clinicians in making precise diagnoses. (Johnson et al., 2023). In this study, two core genes, *OPRM1* and *VIM*, were identified among 96 target genes using LASSO regression and SVM algorithm analyses. Furthermore, these two core genes demonstrated superior predictive efficacy in the ROC curves for diagnostic validation, suggesting that *OPRM1* and *VIM* have the potential to predict the development of TS disease. The reliable biomarkers may contribute to accurate diagnose for TS in future, helping for timely and effective treatment. (Xi et al., 2022).

The involvement of immunological pathways is observed in various neurodevelopmental processes such as the neural circuits refinement and formation. However, there is still a lack of information on the efficacy of interventions targeting immune system modulation. (e.g., through environmental modifications) for treating disorders such as TS. (Martino et al., 2020). It is believed that immune mechanisms might function in the developmental changes that underlie the behavioral anomalies seen in TS. (Hsu et al., 2021a; Martino et al., 2015). The *post mortem* studies and animal models have suggested that microglia played vital role in the crosstalk between the neural and immune systems. Clinical research investigating immune cell subpopulations, cytokine and immunoglobulin levels and etc., have indicated the systemic immune response overactivity. The evidence indicated the disease mechanisms in TS, akin to other neurodevelopmental disorders such as autism, could involve impaired communication between the neural and immune systems. Consequently, this may result in changes in the development of brain pathways that control different behavioral aspects and could potentially affect the coordination of stress and immune responses (Martino et al., 2015). A better understanding of the key molecular pathways influencing neuroimmune interactions in TS can facilitate the discovery of effective biomarkers and more personalized therapeutic approaches. Our findings reveal elevated neutrophil levels in the TS disease cohort compared to controls, consistent with existing literature. Previous studies have observed increased neutrophil-lymphocyte ratios (NLR) across multiple psychiatric conditions. This suggests that abnormal NLR may indicate a general pathological brain process rather than being disorder-specific. Such results support the neuroinflammation hypothesis in psychiatric etiology. Future investigations should explore the links between NLR and specific diagnostic and behavioral constructs to further elucidate these relationships (Bhikram and Sandor, 2022). Our immune infiltration analysis confirmed that the core genes are involved in regulating the immune microenvironment. This results is consistent with the previous section in which we found that TNF and IL-17 signaling pathways mainly involved in YXT. Investigations into TS reveal heightened inflammation indicators. Notably, tumor necrosis factor-alpha and various interleukins (e.g., IL-1β through IL-17) show increased presence. Abnormal cytokine levels can mimic TS symptoms, suggesting a potential link between this condition and chronic inflammatory processes (Leckman et al., 2005; Hsu et al., 2021b).

Immune disorders have been implicated in the progression of TS, but no specific targets was reported for treating TS by modulating the immune system. This may explain why immune system modulators are not included in the drugs used for the clinical treatment of TS. As a complementary finding, our results suggest that *OPRM1* and *VIM* could serve as potential drug targets to regulate the expression levels of immune inhibitors and immune simulators, thereby improving the immune microenvironment. Immune simulators related to *OPRM1* include *TNFRSF13B*, while immune inhibitors include *KIR2DL3* and *KIR2DL1*. Immune simulators related to *VIM* include *CXCR4* and *CD48*, while immune inhibitors include *TNFSF9* and *CD276*. The specific roles of these immune-related genes in TS warrant further exploration. In conclusion, this study provides evidence that immune regulation participated in the mechanism of action of YXT in treating TS and suggests that *OPRM1* and *VIM* could be potential targets for regulating the immune microenvironment.

*OPRM1* and *VIM*, as core genes, can not only serve as biomarkers and drug targets for TS but also provide a research entrance for further discovery of TS-related molecular mechanisms. Existing studies have shown a greater inclination towards finding evidence that *OPRM1* affects TS compared to *VIM*. (Depienne et al., 2019; Widomska et al., 2023). In fact, a TS cohort was screened for additional variants in *OPRM1* using Sanger sequencing, which identified eight rare variants. (Tchalova et al., 2021). Interestingly, all *OPRM1* variations were inherited from a parent without symptoms, suggesting that variants in opioid receptors may be susceptibility factors for TS. (Tchalova et al., 2021). Furthermore, frequent variants of *OPRM1* have been correlated with impulse control disorders in Parkinson patients recently. (Cormier-Dequaire et al., 2018). The discovery supported that mutations in opioid receptors play a role in impulsive disorders, which are often comorbid with TS (Paschou et al., 2013; Elamin et al., 2013). A recent study aimed to construct a molecular landscape of TD, which revealed signs of increased expression of candidate genes involved in TD in four regions of the brain and the pituitary gland.

The landscape of TS offered opportunity to underly molecular mechanism of TS, and provided potential targets for drug intervention in Tic Disorders (TD). *OPRM1*, *CX3CL1-CX3CR1*, *NAALAD2*, *FLT3* are examples of these potential targets. (Widomska et al., 2023). In line with previous research, we have identified *OPRM1* and *VIM* as core genes in TS and established a network of target pathways. Previous studies have shown that *OPRM1* exhibits regional specificity in TD, being highly expressed in the TD patient brains but specifically decreased in the striatum *postmortem*. (Lennington et al., 2016). We also investigated the possible signaling pathways related with *OPRM1* and *VIM*, and conducted enrichment analyses using GO and KEGG. Additionally, we explored potential mechanisms for the transcriptional regulation. As a result, 59 pairs of mRNA-miRNA interactions related to *OPRM1* and 34 pairs related to *VIM* were predicted. Furthermore, we found remarkable association between the *OPRM1* and *VIM* expression, and other TS-related genes, including *MAPT* and *MAPT*. These findings provide evidence supporting the use of *OPRM1* and *VIM* as biomarkers and drug targets in the context of TS.

Gene expression regulation heavily relies on transcription factors (Dec. Erga et al., 2018). We utilized RcisTarget to identify significant binding motifs and their associated transcription factors for *OPRM1* and *VIM* genes. This approach allows prediction of potential binding sites through motif sequence extraction, enabling further investigation of underlying molecular mechanisms. (Kuret et al., 2022). MicroRNAs (miRNAs) are short non-coding RNA sequences, spanning 21-25 nucleotides, that can bind to the 3′ UTR of target mRNAs, resulting in their degradation or translation suppression. (He and Hannon, 2004). Our mRNA-miRNA regulatory network analysis revealed miR-155, miR-204, miR-124, and miR-129-5p as the most highly connected among the four hub genes. Research has shown that miRNAs can influence inflammatory signaling, potentially leading to uncontrolled neuroinflammation and associated pathologies like Tourette Syndrome (TS) (Rizzo et al., 2015). For instance, miR-155 acts as a pro-inflammatory mediator in the central nervous system and plays a crucial role in neuroinflammatory disorders such as multiple sclerosis and Alzheimer’s disease (Ghafouri-Fard et al., 2021). miR-124, while predominantly expressed in the brain, is also found in various human and animal tissues. It contributes to the pathogenesis of several disorders, and its abnormal expression has been linked to various neurological conditions due to its vital role in nervous system development (Zingale et al., 2021). Targeting miRNA expression offers a promising therapeutic avenue for addressing pathological neuroinflammation. In this research, we constructed an mRNA-miRNA regulatory network of hub genes to identify various miRNAs that may influence TS development and progression.

Network pharmacology is a promising technique that offers advantages such as low cost and high efficiency (Luo et al., 2020; Zhou et al., 2020). However, it still has some limitations. This study identifies three main limitations. Firstly, the study’s objectives were sourced from diverse databases, each with distinct areas of emphasis. It is crucial to acknowledge that conducting an integrated analysis of multiple databases carries inherent risks owing to their inherent dissimilarities. Secondly, additional foundational experimental and clinical investigations can be performed to corroborate the precision of the results. Network pharmacology theoretically analyzes the material basis of TCM for treating a certain disease, but ignores the feasibility from prediction to realization. For example, there is a wide variety of ingredients and their contents vary greatly in herbs. Network pharmacology cannot take into account the reduction in likelihood by lower ingredient content. In fact, some ingredients, although known to be effective, cannot be purified because of their extremely low content or cannot be industrially synthesized because of their complex structure. Thirdly, the qualitative phase of network pharmacology research on TCM formula investigation still focuses primarily on exploring novel targets and understanding the drug’s mechanism. However, it is crucial to establish a relationship between the dosage of the medication and the specific ailment.

## 5 Conclusion

TS, a disorder with multiple pathogenic mechanisms and undefined pathological targets, is often associated with obsessive-compulsive disorder, sleep disorders, mood disorders, and other psychobehavioral problems. This may explain why existing drugs are not effective in treating TS. YXD, a compound prescription of Traditional Chinese Medicines, has multiple targets and mechanisms of action, providing more satisfactory clinical efficacy. Network pharmacology was utilized to map the component-target-pathway network of YXD for the treatment of TS. Through this approach, we identified two core genes, *OPRM1* and *VIM*, and predicted their molecular mechanisms in affecting TS through immune infiltration and transcriptional regulation. Based on the reported evidence, *OPRM1* appears to be a plausible biomarker for TS. The active ingredients, targets and molecular mechanisms were elucidated to some extent, which will promote the clinical application of YXD. In future applications of YXD in clinical therapy or drug development, it is important to consider the challenges posed by the content and structure of the individual active ingredients.

## Data Availability

The data used in our study is publicly available. TCMSP Database: Data can be found at https://old.tcmsp-e.com/tcmsp.php. OMIM Database: Data can be found at https://www.omim.org/. Genecard Database: Data can be found at https://www.genecards.org/. GEO Database: Data can be found at https://www.ncbi.nlm.nih.gov/geo/. TS Dataset GSE30470: The specific dataset can be accessed at https://www.ncbi.nlm.nih.gov/geo/query/acc.cgi?acc=GSE30470.

## References

[B1] Bhikram T. Sandor P. (2022). Neutrophil-lymphocyte ratios as inflammatory biomarkers in psychiatric patients. Behav Immun 105, 237–246. 10.1016/j.bbi.2022.07.006 35839998

[B2] Billnitzer A. Jankovic J. (2020). Current anagement of ics and ourette yndrome: ehavioral, harmacologic, and urgical reatments. Neurotherapeutics 17, 1681–1693. 10.1007/s13311-020-00914-6 32856174 PMC7851278

[B3] Brady M. V. Mariani J. Koca Y. Szekely A. King R. A. Bloch M. A.-O. (2022). Mispatterning and interneuron deficit in Tourette Syndrome basal ganglia organoids. Molecular psychiatry, 1476–5578. 10.1038/s41380-022-01880-5 PMC9949887 36447010

[B4] Buse J. Schoenefeld K Fau - Münchau A. Münchau A Fau - Roessner V. Roessner V. (2013). Neuromodulation in Tourette syndrome: dopamine and beyond. Neurosci Biobehav Rev, 1873–7528. 10.1016/j.neubiorev.2013.03.012 23085211

[B5] Cavanna A. E. Ganos C. Hartmann A. Martino D. Pringsheim T. Seri S. (2020). The cognitive neuropsychiatry of Tourette syndrome. Cogn Neuropsychiatry, 1464–0619. 10.1080/13546805.2020.1760812 32372718

[B6] Chen C. W. Hsueh C. W. Chung C. H. Wang H. S. Chang H. J. Chien W. C. (2020). The association between tic medication therapy and psychiatric comorbidities among patients with Tourette syndrome: national population-based study in Taiwan. Brain Dev, 1872–7131. 10.1016/j.braindev.2020.01.002 32029325

[B7] Chou C. Y. Agin-Liebes J. Kuo S. H. (2023). Emerging therapies and recent advances for Tourette syndrome. Heliyon 9, 2405–8440. 10.1016/j.heliyon.2023.e12874 PMC9860289 36691528

[B8] Cormier-Dequaire F. Bekadar S. Anheim M. Lebbah S. Pelissolo A. Krack P. (2018). Suggestive association between OPRM1 and impulse control disorders in Parkinson's disease. Electronic 33, 1878–1886. 10.1002/mds.27519 30444952

[B9] Dec. Erga A. H. Dalen I. Ushakova A. Chung J. Tzoulis C. Tysnes O. B. (2018). Dopaminergic and pioid athways ssociated with mpulse ontrol isorders in Parkinson's isease. Front. Neurosci 15, 1664–2295. 10.3389/fnins.2021.654238 PMC5835501 29541058

[B10] Depienne C. Ciura S. Trouillard O. Bouteiller D. Leitão E. Nava C. (2019). Association of are enetic ariants in pioid eceptors with ourette yndrome. LID 9, 2160–8288. 10.5334/tohm.464

[B11] Domènech L. Cappi C. Halvorsen M. A.-O. (2021). Genetic architecture of Tourette syndrome: Our current understanding. J. Genet. Psychol. 10.1017/S0033291721000234 PMC10763736 33612126

[B12] Eapen V. Cavanna A. E. Robertson M. M. (2016a). Social impact and quality of life in Tourette syndrome. J. Behav. Health. 10.3389/fpsyt.2016.00097 PMC4893483 27375503

[B13] Eapen V. Snedden C. Črnčec R. (2016b). Tourette syndrome, co-morbidities, and quality of life. Clin. Psychol. Rev. 10.1177/0004867415594429 26169656

[B14] Efron D. Dale R. C. (2018). Tics and Tourette syndrome. Neuropsychiatr. dis. treat. 10.1111/jpc.14165 30294996

[B15] Elamin I. Edwards Mj Fau - Martino D. Martino (2013). Immune dysfunction in Tourette syndrome. J. Immunol. Res. 10.3233/BEN-120295 PMC5214461 23187145

[B16] Felling R. J. Singer H. S. (2011). Neurobiology of syndrome: current status and need for further investigation. J Neurosci 31, 1529–2401. 10.1523/JNEUROSCI.0150-11.2011 PMC6703258 21880899

[B17] First M. B. (2012). “Diagnostic and statistical manual of mental disorders,” in linical utility. Editors Cavanna A. E. Termine C. 5th Edn (Washington, DC: Tourette syndrome), 1539–736. 10.1097/NMD.0b013e3182a2168a

[B18] Ghafouri-Fard S. Shoorei H. Bahroudi Z. Abak A. Majidpoor J. Taheri M. (2021). An update on the role of miR-124 in the pathogenesis of human disorders. Biomed Pharmacother 135, 111198. 10.1016/j.biopha.2020.111198 33412388

[B19] Gilbert D. L. Dubow J. S. Cunniff T. M. Wanaski S. P. Atkinson S. D. Mahableshwarkar A. R. (2024). Innovative therapies for Tourette syndrome: a trial. Pediatrics, e2022059574. 10.1542/peds.2022-059574 36628546

[B20] Hartmann A. Worbe Y. (2018). Tourette syndrome: clinical spectrum, mechanisms and personalized treatments. (1473–6551. 10.1097/WCO.0000000000000575 29746399

[B21] He L. Hannon G. J. MRAs (2004). MicroRNAs: small RNAs with a big role in gene regulation. Nat Rev Genet 5 (7), 522–531. 10.1038/nrg1379 15211354

[B22] Hsu C. J. Wong L. C. Lee W. T. (2021a). Immunological ysfunction in ourette yndrome and elated isorders. Int. J. Mol. Sci. 22, 853. LID - 10.3390/ijms22020853 [doi] LID - 853. (1422-0067). 10.3390/ijms22020853 33467014 PMC7839977

[B23] Hsu C. J. Wong L. C. Lee W. T. (2021b). Immunological ysfunction in ourette yndrome and elated isorders. Int J Mol Sci 22 (2), 853. 10.3390/ijms22020853 33467014 PMC7839977

[B24] Johnson K. A. Worbe Y. Foote K. D. Butson C. R. Gunduz A. Okun M. S. (2023). Tourette syndrome: clinical features. 1474–4465. 10.1016/S1474-4422(22)00303-9 PMC10958485 36354027

[B25] Kattner A. A. (2022). “What makes tics tick? Insights into tourette syndrome,” in Neuroscience and Biobehavioral Reviews. New York, NY: Elsevier. 10.1016/j.bj.2022.04.004 PMC9250088 35460927

[B26] Kim D. D. Barr A. M. Chung Y. Yuen J. W. Y. Etminan M. Carleton B. C. (2018). Antipsychotic-ssociated symptoms of tourette syndrome. A Syst Rev, 1179–1934. 10.1007/s40263-018-0559-8 30121819

[B27] Kuret K. Amalietti A. G. Jones D. M. Capitanchik C. Ule J. (2022). Positional motif analysis reveals the extent of specificity of protein-RNA interactions observed by CLIP. Genome Biol 23 (1), 191. 10.1186/s13059-022-02755-2 36085079 PMC9461102

[B28] Leckman J. F. Katsovich L Fau - Kawikova I. Kawikova I Fau - Lin H. Lin Fau - Zhang H. Zhang H Fau - Krönig H. (2005). Increased serum levels of interleukin-12 and tumor necrosis factor-alpha in Tourette's syndrome. Biol. Psychiatry 57, 667–673. 0006-3223). 10.1016/j.biopsych.2004.12.004 15780855

[B29] Lennington J. B. Coppola G. Kataoka-Sasaki Y. Fernandez T. V. Palejev D. Li Y. (2016). Transcriptome nalysis of the uman triatum in ourette yndrome. Biol Psychiatry 372, 1873–2402. 10.1016/j.biopsych.2014.07.018 PMC4305353 25199956

[B30] Lin W. D. Tsai F. J. Chou I. C. (2022). “Current understanding of the genetics of Tourette syndrome,” in Journal of Medical Genetics. London, UK: BMJ Publishing Group. 10.1016/j.bj.2022.01.008 PMC9250083 35042017

[B31] Liu Z. Li H. Pan S. (2021). Discovery and alidation of ey iomarkers ased on mmune nfiltrates in Alzheimer's isease. Front Genet 12, 658323. 10.3389/fgene.2021.658323 34276768 PMC8281057

[B32] Liu Z. S. Cui Y. H. Sun D. Lu Q. Jiang Y. W. Jiang L. (2020). Current tatus, iagnosis, and reatment ecommendation for ic isorders in China. Front. Psychiatry 11, 1664–0640. 10.3389/fpsyt.2020.00774 PMC7438753 32903695

[B33] Luo T. T. Lu Y. Yan S. K. Xiao X. Rong X. L. Guo J. (2020). Network harmacology in esearch of Chinese edicine ormula: ethodology, pplication and rospective. Chin. J. Integr. Med. 26, 72–80. 10.1007/s11655-019-3064-0 30941682

[B34] Martino D. Johnson I. Leckman J. F. (2020). What oes mmunology ave to o ith ormal rain evelopment and the athophysiology nderlying ourette yndrome and elated europsychiatric isorders? Front. Neurol. 11, 1664–2295. 10.3389/fneur.2020.567407 PMC7525089 33041996

[B35] Martino D. Zis P. Buttiglione M. (2015). “The role of immune mechanisms in Tourette syndrome,” in Brain, Behavior, and Immunity. San Diego, CA: Academic Press. 10.1016/j.brainres.2014.04.027 24845720

[B36] Nilles C. Hartmann A. Roze E. Martino D. Pringsheim T. (2023). Tourette syndrome and other tic disorders of childhood. 10.1016/B978-0-323-98817-9.00002-8 37620085

[B37] Niu B. Xie X. Xiong X. Jiang J. (2022). “Network pharmacology-based analysis of the anti-hyperglycemic active ingredients and experimental validation,” in Pharmacological Research. London, UK: Elsevier. 10.1016/j.compbiomed.2021.104636 34809966

[B38] Ogundele M. O. Ayyash H. F. (2018). Review of the evidence for the management of co-morbid Tic disorders in children and adolescents with attention deficit hyperactivity disorder. World J Clin Pediatr 7, 2219–2808. 10.5409/wjcp.v7.i1.36 PMC5803563 29456930

[B39] Paschou P. Fernandez Tv Fau - Sharp F. Sharp F Fau - Heiman G. A. Heiman Ga Fau - Hoekstra P. J. Hoekstra P. J. (2013). Genetic susceptibility and neurotransmitters in Tourette syndrome. Electronic, 2162–5514. 10.1016/B978-0-12-411546-0.00006-8 PMC4471172 24295621

[B40] Pringsheim T. Holler-Managan Y. Okun M. S. Jankovic J. Piacentini J. Cavanna A. E. (2019a). Comprehensive systematic review summary: reatment of tics in people with Tourette syndrome and chronic tic disorders. Neurology 92, 1526–632X. 10.1212/WNL.0000000000007467 PMC6537130 31061209

[B41] Pringsheim T. Okun M. S. Müller-Vahl K. Martino D. Jankovic J. Cavanna A. E. (2019b). Practice guideline recommendations summary: reatment of tics in people with Tourette syndrome and chronic tic disorders. 10.1212/WNL.0000000000007466 PMC6537133 31061208

[B42] Ramkiran S. Heidemeyer L. Gaebler A. Shah N. J. Neuner I. (2019). Alterations in basal ganglia-cerebello-thalamo-cortical connectivity and whole brain functional network topology in Tourette’s syndrome. NeuroImage: Clinical 24, 101998. 10.1016/j.nicl.2019.101998 31518769 PMC6742843

[B43] Rizzo R. Ragusa M. Barbagallo C. Sammito M. Gulisano M. Calì P. V. (2015). Circulating miRNAs profiles in Tourette syndrome: molecular data and clinical implications. Mol 8, 44. 10.1186/s13041-015-0133-y PMC4513635 26205656

[B44] Seideman Mf Fau - Seideman T. A. Seideman T. A. (2020). A eview of the urrent reatment of ourette yndrome. 10.5863/1551-6776-25.5.401 PMC7337131 32641910

[B45] Set K. K. Warner J. N. (2021). Traditional Chinese medicine (TCM) therapy. J. Altern. Complement. Med.

[B46] Shang L. Wang Y. Li J. Zhou F. Xiao K. Liu Y. (2023). Mechanism of Decoction in the treatment of colorectal cancer based on network pharmacology and experimental validation. J Ethnopharmacol 302, 1872–7573. 10.1016/j.jep.2022.115876 36343798

[B47] Szejko N. (2022). Chapter Four - update and recent progress in the neurobiology of Tourette syndrome. In International review of movement disorders, Lavoie M. E. Cavanna A. E. 3; Academic Press, United States 131–158.

[B48] Tchalova K. Sadikaj G. Moskowitz D. S. Zuroff D. C. Bartz J. A. (2021). Variation in the μ-opioid receptor gene (OPRM1) and experiences of felt security in response to a romantic partner's quarrelsome behavior. Mol Psychiatry 26, 1476–5578. 10.1038/s41380-019-0600-4 31772303

[B49] Tsetsos F. A.-O. X. Yu D. Sul J. H. Huang A. Y. Illmann C. Osiecki L. (2024). Synaptic processes and immune-related pathways implicated in Tourette syndrome. Transl. Psychiatry. 18. 10.1038/s41537-024-00156-7 PMC7814139 33462189

[B50] Widomska J. De Witte W. Buitelaar J. A.-O. Glennon J. A.-O. Poelmans G. (2023). Molecular andscape of ourette's isorder. Int. J. Mol. Sci. 24, 1428. LID - 10.3390/ijms24021428 [doi] LID - 1428. (1422-0067 (Electronic)). From. 10.3390/ijms24021428 36674940 PMC9865021

[B51] Xi L. A.-O. Zhou F. A.-O. X. Ji W. A.-O. Zhu W. A-O. X. Ruan J. A.-O. Zhang Y. A.-O. (2022). Potential lasma etabolic iomarkers of ourette yndrome iscovery ased on ntegrated ontargeted and argeted etabolomics creening lasma etabolic iomarkers of TS. Evid Based Complement Alternat Med 2022, 1741–427X. 10.1155/2022/5080282 PMC9894715 36742270

[B52] Xiao L. J. Tao R. (2017). Traditional Chinese edicine (TCM) therapy, 0065–2598. 10.1007/978-981-10-5562-1_13 29098677

[B53] Zhou Z. A-O. Chen B. Chen S. Lin M. Chen Y. Jin S. (2020). Applications of network pharmacology in traditional Chinese medicine research, 2020, 1741–427X. 10.1155/2020/1646905 PMC7042531 32148533

[B54] Zingale V. D. Gugliandolo A. Mazzon E. (2021). MiR-155: n mportant egulator of euroinflammation. Int J Mol Sci 23 (1), 90. 10.3390/ijms23010090 35008513 PMC8745074

